# (5*Z*)-5-(2-Methyl­benzyl­idene)-3-phenyl-2-thioxo-1,3-thia­zolidin-4-one

**DOI:** 10.1107/S1600536809044304

**Published:** 2009-10-31

**Authors:** Durre Shahwar, M. Nawaz Tahir, Muhammad Asam Raza, Bushra Iqbal

**Affiliations:** a Department of Chemistry, Government College University, Lahore, Pakistan; b Department of Physics, University of Sargodha, Sargodha, Pakistan

## Abstract

In the title compound, C_17_H_13_NOS_2_, the heterocyclic ring is oriented at a dihedral angle of 74.43 (5)° with respect to the anilinic benzene ring and at a dihedral angle of 17.31 (9)° with respect to phenyl ring. An intra­molecular C—H⋯S inter­action occurs, resulting in an *S*(6) ring. In the crystal, the packing is consolidated by C—H⋯π inter­actions and possible very weak aromatic π–π stacking [centroid–centroid separation = 4.025 (1) Å].

## Related literature

For related structures, see: Linden *et al.* (1999 ▶); Shahwar *et al.* (2009*a* ▶,*b* ▶,*c* ▶). For graph-set theory, see: Bernstein *et al.* (1995 ▶).
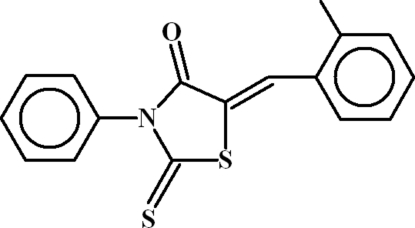


## Experimental

### 

#### Crystal data

C_17_H_13_NOS_2_
*M* *_r_* = 311.40
Monoclinic, 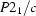

*a* = 9.8317 (4) Å
*b* = 16.6317 (6) Å
*c* = 9.3865 (4) Å
β = 93.541 (2)°
*V* = 1531.93 (11) Å^3^
*Z* = 4
Mo *K*α radiation
μ = 0.35 mm^−1^
*T* = 296 K
0.40 × 0.30 × 0.18 mm

#### Data collection

Bruker Kappa APEXII CCD diffractometer
Absorption correction: multi-scan (*SADABS*; Bruker, 2005 ▶) *T*_min_ = 0.879, *T*_max_ = 0.941
17261 measured reflections
3807 independent reflections
2879 reflections with *I* > 2σ(*I*)
*R*_int_ = 0.028

#### Refinement

*R*[*F* ^2^ > 2σ(*F* ^2^)] = 0.036
*wR*(*F* ^2^) = 0.104
*S* = 1.01
3807 reflections
191 parameters
H-atom parameters constrained
Δρ_max_ = 0.24 e Å^−3^
Δρ_min_ = −0.20 e Å^−3^

### 

Data collection: *APEX2* (Bruker, 2007 ▶); cell refinement: *SAINT* (Bruker, 2007 ▶); data reduction: *SAINT*; program(s) used to solve structure: *SHELXS97* (Sheldrick, 2008 ▶); program(s) used to refine structure: *SHELXL97* (Sheldrick, 2008 ▶); molecular graphics: *ORTEP-3* (Farrugia, 1997 ▶) and *PLATON* (Spek, 2009 ▶); software used to prepare material for publication: *WinGX* (Farrugia, 1999 ▶) and *PLATON*.

## Supplementary Material

Crystal structure: contains datablocks global, I. DOI: 10.1107/S1600536809044304/hb5181sup1.cif

Structure factors: contains datablocks I. DOI: 10.1107/S1600536809044304/hb5181Isup2.hkl

Additional supplementary materials:  crystallographic information; 3D view; checkCIF report

## Figures and Tables

**Table 1:** Hydrogen-bond geometry (Å, °)

*D*—H⋯*A*	*D*—H	H⋯*A*	*D*⋯*A*	*D*—H⋯*A*
C16—H16⋯S1	0.93	2.52	3.2197 (19)	133
C17—H17*C*⋯CgC^i^	0.96	2.72	3.569 (2)	148

Symmetry code: (i) 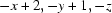. *CgC* is the centroid of C11–C16 benzene ring.

**Fig. 1.**
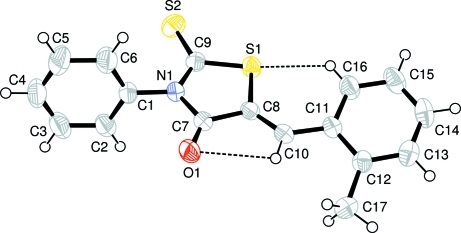
View of (I) with displacement ellipsoids drawn at the 50% probability level. The dotted line represents the intramolecular H-bond.

## supplementary crystallographic information

### Comment

The title compound (I, Fig. 1), has been prepared and being reported in continuation of synthesizing various derivatives of rhodanine. In this context we have reported the crystal structure of (II) (5*Z*)-5-(2-Hydroxybenzylidene)-3-phenyl-2-thioxo-1,3-thiazolidin-4-one (Shahwar *et al.*, 2009*a*), (III) (5*Z*)-5-(2-Hydroxybenzylidene)-2-thioxo-1,3-thiazolidin-4-one methanol hemisolvate (Shahwar *et al.*, 2009*b*) and (IV) (5*E*)-5-(4-Hydroxy-3-methoxybenzylidene)-2-thioxo-1,3-thiazolidin- 4-one methanol monosolvate (Shahwar *et al.*, 2009*c*).

The crystal structure of (I) differs from (V) 3-Phenyl-5-(phenylmethylidene)-2-thioxo-1,3-thiazolidin-4-one (Linden *et al.*, 1999) due to attachement of methyl group.

In (I) the heterocyclic ring A (N1/C7/S1/C8/C9), two benzene rings B (C1—C6) and C (C11–C16) are planar with maximum r. m. s. deviations of 0.0047, 0.0074 and 0.0046 Å respectively, from the respective mean square planes. The dihedral angles between A/B, A/C and B/C are 74.43 (5), 17.31 (9) and 59.19 (6)°, respectively. The intramolecular H-bondings of C—H···S (Table 1, Fig. 1) form S(6) ring motif (Bernstein *et al.*, 1995). There exist π···π-interactions between adjacent molecules. The CgA···CgC^i^ and CgC···CgA^i^ [symmetry code: i = 2 - *x*, 1 - *y*, 1 - *z*] have centroid to centroid distance of 4.025 (1) Å, where CgA and CgC are the centroids of rings A and C, respectively. The C–H···π interactions (Table 1) also play role in stabilizing the molecules.

### Experimental

3-Phenyl-2-thioxo-1,3-thiazolidin-4-one (0.419 g, 0.2 mol), 2-Methylbenzaldehyde (0.240 g, 0.2 mol) and K_2_CO_3_ (0.553 g, 0.4 mol) were dissolved in 10 ml distilled water at room temperature. The stirring was continued for 24 h and reaction was monitored by TLC. The precipitates were formed during neutalization of the reaction mixture with 5% HCl. The precipitates were filtered off and washed with saturated solution of NaCl. The crude material obtained was recrystalized in ethyl acetate to affoard yellow prisms of (I).

### Refinement

The H-atoms were positioned geometrically (C–H = 0.93–0.96 Å) and refined as riding with *U*_iso_(H) = *xU*_eq_(C), where *x* = 1.5 for methyl and 1.2 for other H atoms.

### Crystal data

**Table:**

C_17_H_13_NOS_2_	*F*(000) = 648
*M**_r_* = 311.40	*D*_x_ = 1.350 Mg m^−^^3^
Monoclinic, *P*2_1_/*c*	Mo *K*α radiation, λ = 0.71073 Å
Hall symbol: -P 2ybc	Cell parameters from 3807 reflections
*a* = 9.8317 (4) Å	θ = 2.1–28.3°
*b* = 16.6317 (6) Å	µ = 0.35 mm^−^^1^
*c* = 9.3865 (4) Å	*T* = 296 K
β = 93.541 (2)°	Prisms, yellow
*V* = 1531.93 (11) Å^3^	0.40 × 0.30 × 0.18 mm
*Z* = 4	

### Data collection

**Table:**

Bruker Kappa APEXII CCD diffractometer	3807 independent reflections
Radiation source: fine-focus sealed tube	2879 reflections with *I* > 2σ(*I*)
graphite	*R*_int_ = 0.028
Detector resolution: 7.40 pixels mm^-1^	θ_max_ = 28.3°, θ_min_ = 2.1°
ω scans	*h* = −13→12
Absorption correction: multi-scan (*SADABS*; Bruker, 2005)	*k* = −13→22
*T*_min_ = 0.879, *T*_max_ = 0.941	*l* = −12→9
17261 measured reflections	

### Refinement

**Table:**

Refinement on *F*^2^	Primary atom site location: structure-invariant direct methods
Least-squares matrix: full	Secondary atom site location: difference Fourier map
*R*[*F*^2^ > 2σ(*F*^2^)] = 0.036	Hydrogen site location: inferred from neighbouring sites
*wR*(*F*^2^) = 0.104	H-atom parameters constrained
*S* = 1.01	*w* = 1/[σ^2^(*F*_o_^2^) + (0.0498*P*)^2^ + 0.3691*P*] where *P* = (*F*_o_^2^ + 2*F*_c_^2^)/3
3807 reflections	(Δ/σ)_max_ < 0.001
191 parameters	Δρ_max_ = 0.24 e Å^−^^3^
0 restraints	Δρ_min_ = −0.20 e Å^−^^3^

### Special details

**Table:**

Geometry. Bond distances, angles *etc*. have been calculated using the rounded fractional coordinates. All su's are estimated from the variances of the (full) variance-covariance matrix. The cell e.s.d.'s are taken into account in the estimation of distances, angles and torsion angles
Refinement. Refinement of *F*^2^ against ALL reflections. The weighted *R*-factor *wR* and goodness of fit *S* are based on *F*^2^, conventional *R*-factors *R* are based on *F*, with *F* set to zero for negative *F*^2^. The threshold expression of *F*^2^ > σ(*F*^2^) is used only for calculating *R*-factors(gt) *etc*. and is not relevant to the choice of reflections for refinement. *R*-factors based on *F*^2^ are statistically about twice as large as those based on *F*, and *R*- factors based on ALL data will be even larger.

### Fractional atomic coordinates and isotropic or equivalent isotropic displacement parameters (Å^2^)

**Table:**

	*x*	*y*	*z*	*U*_iso_*/*U*_eq_	
S1	0.95527 (4)	0.36087 (3)	0.48338 (4)	0.0408 (1)	
S2	0.81145 (5)	0.25140 (3)	0.66855 (5)	0.0544 (2)	
O1	0.62790 (12)	0.44603 (8)	0.30111 (16)	0.0590 (5)	
N1	0.69362 (13)	0.35311 (8)	0.47353 (15)	0.0379 (4)	
C1	0.55749 (16)	0.32728 (10)	0.49995 (18)	0.0409 (5)	
C2	0.47556 (18)	0.37600 (11)	0.5756 (2)	0.0508 (6)	
C3	0.3467 (2)	0.34908 (14)	0.6034 (3)	0.0648 (8)	
C4	0.3022 (2)	0.27496 (15)	0.5571 (3)	0.0686 (8)	
C5	0.3841 (2)	0.22789 (15)	0.4798 (3)	0.0774 (9)	
C6	0.5136 (2)	0.25369 (12)	0.4493 (3)	0.0644 (8)	
C7	0.71757 (16)	0.41175 (9)	0.37026 (18)	0.0400 (5)	
C8	0.86629 (15)	0.42329 (9)	0.36025 (17)	0.0358 (5)	
C9	0.80674 (16)	0.32015 (9)	0.54449 (17)	0.0374 (5)	
C10	0.91369 (16)	0.47413 (9)	0.26411 (18)	0.0390 (5)	
C11	1.05266 (15)	0.49045 (10)	0.22684 (17)	0.0383 (5)	
C12	1.07841 (16)	0.55752 (10)	0.14107 (17)	0.0393 (5)	
C13	1.20990 (18)	0.56853 (12)	0.0986 (2)	0.0522 (6)	
C14	1.31437 (18)	0.51669 (14)	0.1394 (2)	0.0591 (7)	
C15	1.29056 (18)	0.45192 (13)	0.2249 (2)	0.0580 (7)	
C16	1.16070 (17)	0.43879 (12)	0.2676 (2)	0.0509 (6)	
C17	0.96876 (19)	0.61723 (10)	0.0967 (2)	0.0500 (6)	
H2	0.50600	0.42618	0.60750	0.0609*	
H3	0.28976	0.38161	0.65410	0.0777*	
H4	0.21635	0.25676	0.57835	0.0823*	
H5	0.35298	0.17800	0.44709	0.0929*	
H6	0.56916	0.22185	0.39589	0.0773*	
H10	0.84702	0.50379	0.21293	0.0468*	
H13	1.22796	0.61221	0.04085	0.0626*	
H14	1.40133	0.52561	0.10891	0.0709*	
H15	1.36121	0.41725	0.25365	0.0695*	
H16	1.14447	0.39461	0.32487	0.0611*	
H17A	1.00596	0.65805	0.03813	0.0751*	
H17B	0.93458	0.64157	0.18005	0.0751*	
H17C	0.89582	0.59014	0.04357	0.0751*	

### Atomic displacement parameters (Å^2^)

**Table:**

	*U*^11^	*U*^22^	*U*^33^	*U*^12^	*U*^13^	*U*^23^
S1	0.0330 (2)	0.0478 (2)	0.0411 (2)	0.0007 (2)	−0.0014 (2)	0.0040 (2)
S2	0.0639 (3)	0.0516 (3)	0.0477 (3)	−0.0025 (2)	0.0028 (2)	0.0119 (2)
O1	0.0342 (6)	0.0622 (8)	0.0804 (10)	0.0084 (6)	0.0030 (6)	0.0248 (7)
N1	0.0321 (7)	0.0371 (7)	0.0450 (8)	−0.0012 (5)	0.0062 (5)	0.0000 (6)
C1	0.0342 (8)	0.0439 (9)	0.0451 (9)	−0.0024 (7)	0.0059 (7)	0.0020 (7)
C2	0.0418 (9)	0.0535 (10)	0.0578 (11)	0.0034 (8)	0.0089 (8)	−0.0037 (9)
C3	0.0432 (11)	0.0808 (15)	0.0722 (14)	0.0103 (10)	0.0183 (10)	0.0057 (12)
C4	0.0382 (10)	0.0817 (15)	0.0866 (16)	−0.0095 (10)	0.0095 (10)	0.0170 (13)
C5	0.0572 (13)	0.0668 (14)	0.109 (2)	−0.0241 (11)	0.0110 (13)	−0.0107 (14)
C6	0.0501 (11)	0.0584 (12)	0.0864 (16)	−0.0107 (9)	0.0177 (11)	−0.0182 (11)
C7	0.0341 (8)	0.0369 (8)	0.0495 (9)	0.0031 (6)	0.0066 (7)	0.0013 (7)
C8	0.0321 (8)	0.0346 (8)	0.0408 (8)	0.0034 (6)	0.0023 (6)	−0.0016 (6)
C9	0.0396 (8)	0.0364 (8)	0.0363 (8)	−0.0013 (6)	0.0035 (6)	−0.0048 (6)
C10	0.0330 (8)	0.0390 (8)	0.0451 (9)	0.0053 (6)	0.0026 (7)	0.0011 (7)
C11	0.0330 (8)	0.0445 (8)	0.0374 (8)	0.0006 (7)	0.0025 (6)	−0.0017 (7)
C12	0.0378 (8)	0.0421 (8)	0.0379 (9)	−0.0053 (7)	0.0022 (7)	−0.0042 (7)
C13	0.0482 (10)	0.0555 (11)	0.0535 (11)	−0.0121 (8)	0.0076 (8)	0.0006 (9)
C14	0.0343 (9)	0.0815 (14)	0.0623 (12)	−0.0096 (9)	0.0106 (8)	−0.0051 (11)
C15	0.0336 (9)	0.0813 (14)	0.0589 (12)	0.0108 (9)	0.0014 (8)	0.0055 (10)
C16	0.0383 (9)	0.0640 (12)	0.0508 (10)	0.0069 (8)	0.0060 (8)	0.0122 (9)
C17	0.0530 (10)	0.0416 (9)	0.0556 (11)	−0.0013 (8)	0.0043 (8)	0.0059 (8)

### Geometric parameters (Å, °)

**Table:**

S1—C8	1.7476 (16)	C12—C13	1.388 (2)
S1—C9	1.7389 (16)	C12—C17	1.506 (2)
S2—C9	1.6306 (16)	C13—C14	1.377 (3)
O1—C7	1.205 (2)	C14—C15	1.372 (3)
N1—C1	1.442 (2)	C15—C16	1.379 (2)
N1—C7	1.405 (2)	C2—H2	0.9300
N1—C9	1.375 (2)	C3—H3	0.9300
C1—C2	1.371 (2)	C4—H4	0.9300
C1—C6	1.373 (3)	C5—H5	0.9300
C2—C3	1.384 (3)	C6—H6	0.9300
C3—C4	1.370 (3)	C10—H10	0.9300
C4—C5	1.364 (3)	C13—H13	0.9300
C5—C6	1.390 (3)	C14—H14	0.9300
C7—C8	1.483 (2)	C15—H15	0.9300
C8—C10	1.341 (2)	C16—H16	0.9300
C10—C11	1.457 (2)	C17—H17A	0.9600
C11—C12	1.408 (2)	C17—H17B	0.9600
C11—C16	1.401 (2)	C17—H17C	0.9600
			
C8—S1—C9	93.05 (7)	C13—C14—C15	120.20 (17)
C1—N1—C7	121.49 (13)	C14—C15—C16	119.26 (18)
C1—N1—C9	121.99 (13)	C11—C16—C15	121.57 (18)
C7—N1—C9	116.49 (13)	C1—C2—H2	121.00
N1—C1—C2	119.62 (15)	C3—C2—H2	121.00
N1—C1—C6	118.77 (15)	C2—C3—H3	120.00
C2—C1—C6	121.60 (16)	C4—C3—H3	120.00
C1—C2—C3	118.72 (18)	C3—C4—H4	120.00
C2—C3—C4	120.6 (2)	C5—C4—H4	120.00
C3—C4—C5	119.9 (2)	C4—C5—H5	120.00
C4—C5—C6	120.7 (2)	C6—C5—H5	120.00
C1—C6—C5	118.4 (2)	C1—C6—H6	121.00
O1—C7—N1	123.48 (15)	C5—C6—H6	121.00
O1—C7—C8	126.56 (15)	C8—C10—H10	115.00
N1—C7—C8	109.96 (13)	C11—C10—H10	115.00
S1—C8—C7	109.66 (11)	C12—C13—H13	119.00
S1—C8—C10	129.72 (12)	C14—C13—H13	119.00
C7—C8—C10	120.60 (14)	C13—C14—H14	120.00
S1—C9—S2	121.42 (10)	C15—C14—H14	120.00
S1—C9—N1	110.83 (11)	C14—C15—H15	120.00
S2—C9—N1	127.74 (12)	C16—C15—H15	120.00
C8—C10—C11	130.48 (15)	C11—C16—H16	119.00
C10—C11—C12	119.31 (14)	C15—C16—H16	119.00
C10—C11—C16	121.79 (15)	C12—C17—H17A	109.00
C12—C11—C16	118.82 (14)	C12—C17—H17B	109.00
C11—C12—C13	118.18 (15)	C12—C17—H17C	109.00
C11—C12—C17	122.00 (14)	H17A—C17—H17B	109.00
C13—C12—C17	119.81 (15)	H17A—C17—H17C	109.00
C12—C13—C14	121.96 (18)	H17B—C17—H17C	109.00
			
C9—S1—C8—C7	0.70 (12)	C3—C4—C5—C6	1.1 (4)
C9—S1—C8—C10	−177.47 (16)	C4—C5—C6—C1	0.6 (4)
C8—S1—C9—S2	179.23 (11)	O1—C7—C8—S1	179.38 (15)
C8—S1—C9—N1	−0.06 (13)	O1—C7—C8—C10	−2.3 (3)
C7—N1—C1—C2	−75.8 (2)	N1—C7—C8—S1	−1.15 (16)
C7—N1—C1—C6	104.8 (2)	N1—C7—C8—C10	177.21 (14)
C9—N1—C1—C2	106.26 (19)	S1—C8—C10—C11	3.5 (3)
C9—N1—C1—C6	−73.2 (2)	C7—C8—C10—C11	−174.47 (16)
C1—N1—C7—O1	2.6 (2)	C8—C10—C11—C12	−168.06 (17)
C1—N1—C7—C8	−176.90 (14)	C8—C10—C11—C16	15.4 (3)
C9—N1—C7—O1	−179.33 (16)	C10—C11—C12—C13	−175.47 (16)
C9—N1—C7—C8	1.18 (19)	C10—C11—C12—C17	5.4 (2)
C1—N1—C9—S1	177.41 (12)	C16—C11—C12—C13	1.1 (2)
C1—N1—C9—S2	−1.8 (2)	C16—C11—C12—C17	−178.04 (16)
C7—N1—C9—S1	−0.66 (17)	C10—C11—C16—C15	176.08 (17)
C7—N1—C9—S2	−179.90 (13)	C12—C11—C16—C15	−0.5 (3)
N1—C1—C2—C3	−178.13 (18)	C11—C12—C13—C14	−0.8 (3)
C6—C1—C2—C3	1.3 (3)	C17—C12—C13—C14	178.36 (17)
N1—C1—C6—C5	177.6 (2)	C12—C13—C14—C15	−0.2 (3)
C2—C1—C6—C5	−1.8 (3)	C13—C14—C15—C16	0.9 (3)
C1—C2—C3—C4	0.5 (3)	C14—C15—C16—C11	−0.6 (3)
C2—C3—C4—C5	−1.6 (4)		

### Hydrogen-bond geometry (Å, °)

**Table:**

*D*—H···*A*	*D*—H	H···*A*	*D*···*A*	*D*—H···*A*
C16—H16···S1	0.93	2.52	3.2197 (19)	133
C17—H17C···CgC^i^	0.96	2.72	3.569 (2)	148

Symmetry codes: (i) −*x*+2, −*y*+1, −*z*.
